# Machine Learning-Based Figure of Merit Model of SIPOS Modulated Drift Region for U-MOSFET

**DOI:** 10.3390/mi15030411

**Published:** 2024-03-19

**Authors:** Zhen Cao, Qi Sun, Chuanfeng Ma, Biao Hou, Licheng Jiao

**Affiliations:** 1 School of Artificial Intelligence, Xidian University, Xi’an 710126, China; qi_sun0328@163.com (Q.S.); machuanfeng1021@163.com (C.M.); vcodec@163.com (B.H.); lchjiao@mail.xidian.edu.cn (L.J.); 2 Hangzhou Research Institute of Technology, Xidian University, Hangzhou 311231, China

**Keywords:** superjunction, UMOSFET, machine learning, Silicon limit

## Abstract

This paper presents a machine learning-based figure of merit model for superjunction (SJ) U-MOSFET (SSJ-UMOS) with a modulated drift region utilizing semi-insulating poly-crystalline silicon (SIPOS) pillars. This SJ drift region modulation is achieved through SIPOS pillars beneath the trench gate, focusing on optimizing the tradeoff between breakdown voltage (BV) and specific ON-resistance (*R_ON_*_,*sp*_). This analytical model considers the effects of electric field modulation, charge-coupling, and majority carrier accumulation due to additional SIPOS pillars. Gaussian process regression is employed for the figure of merit (*FOM* = *BV*^2^/*R_ON_*_,*sp*_) prediction and hyperparameter optimization, ensuring a reasonable and accurate model. A methodology is devised to determine the optimal BV-*R_ON_*_,*sp*_ tradeoff, surpassing the SJ silicon limit. The paper also delves into a discussion of optimal structural parameters for drift region, oxide thickness, and electric field modulation coefficients within the analytical model. The validity of the proposed model is robustly confirmed through comprehensive verification against TCAD simulation results.

## 1. Introduction

Power MOSFETs play a crucial role in power management and energy conversion systems. The superjunction (SJ) theory, utilizing a vertical P-N junction in the drift region, has been widely adopted in the design of vertical discrete power MOSFETs rated from 300 V to 1000 V. This approach achieves notably low specific ON resistance (*R_ON_*_,*sp*_) and high breakdown voltage (BV), surpassing the conventional MOSFET silicon limit defined by *R_ON_*_,*sp*_ = 8.3 × 10^−9^ BV^2.5^ [1]. To further optimize performance, integrating a deep trench and an extended gate offers potential *R_ON_*_,*sp*_ reduction by minimizing device pitch and inducing an accumulation layer [2,3,4]. However, this improvement is hindered by the diminishing electric field (E-field) beneath the trench, and blocking voltage faces limitations due to charge balance issues [5,6].

Several strategies have been proposed to tackle this issue. One approach suggests the use of high-K (HK) dielectric in the drift region, as seen in prior studies [6,7,8]. However, the distribution of the E-field in the drift region is significantly affected by the presence of HK dielectric materials, making the complete optimization of the device’s overall E-field challenging. Another method involves enhancing the BV in UMOS by combining high aspect ratio trenches with high-resistance semi-insulating poly-crystalline silicon (SIPOS) structures [9]. This innovative combination offers UMOS the potential to achieve high BV while maintaining an ultra-low *R_ON_*_,*sp*_.

Recent research has seen a surge in innovative approaches using machine learning techniques for device modeling and optimization [10,11,12,13,14,15,16]. For example, Klemme [12] developed a machine learning method for accurately predicting the transfer characteristics of negative-capacitance FinFET devices. Wang [13] improved an artificial neural network (ANN) model for general transistors by enhancing data pre-processing. Xu [14] introduced a machine-learning regression approach for single-electron transistors (SETs), training a neural network to effectively model SET pulse currents. These studies collectively illuminate the diverse applications of machine learning in device modeling and performance optimization. Zhang [15] proposed a concise modeling method for collaborative optimization and path searching in advanced design techniques using machine learning. Mehta [16] demonstrated the possibility of predicting full transistor current–voltage (IV) and capacitance–voltage (CV) curves using machines trained by technology computer-aided design (TCAD) generated data. These studies collectively illuminate the diverse applications of machine learning in device modeling and performance optimization.

**Figure 1:**
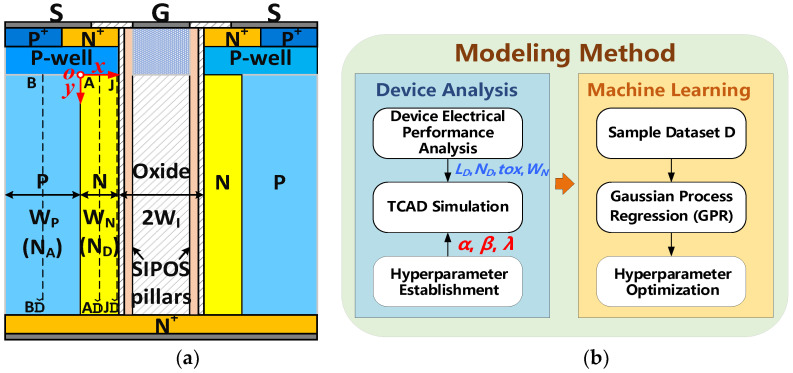
(**a**) Schematic cross-sectional view of SSJ-UMOS, (**b**) machine learning-based modeling methods.

This paper presents a physics-informed and machine learning-based model of the SIPOS (S) pillar-modulated structure in superjunction (SJ) UMOS (SSJ-UMOS), as depicted in Figure 1a,b. The explicit analytical model, grounded in Poisson’s solution, includes the E-field modulation effect, potential distributions, and charge-coupling effects. The model is constructed through a two-step process. Initially, it is derived using closed-form analytical expressions, incorporating Poisson’s solution to capture the basic physical mechanism of the device. Subsequently, machine learning techniques, such as Gaussian process regression (GPR), are employed for the figure of merit (*FOM* = *BV*^2^/*R_ON_*_,*sp*_) prediction and hyperparameter optimization, fine-tuning the model parameters for optimal performance. This combined approach ensures an accurate representation of device behavior, refining predictions of characteristics like the optimal BV-*R_ON_*_,*sp*_ tradeoff, and surpassing the SJ silicon limit [17,18,19]. This hybrid modeling strategy synergizes analytical and machine learning methodologies, yielding a robust and precise device model. The analytical approach of this model can guide the optimization design for MOSFET devices with SIPOS E-field modulation.

## 2. Working Principle and Machine Learn Based Analytical Model

### 2.1. Charge-Coupling Effect of SIPOS Modulated Drift Region

Ref. [19] provides the revised optimum doping concentration (*N_D_*_,*SJ*_) for the N-pillar of the conventional SJ as

(1) $$N_{D,SJ}=2E_{CU}\varepsilon_{Si}/qW_{N}$$

where *W_N_* is the width of the N-pillar, and *E_CU_* is the critical E-field for breakdown with a uniform distribution. In contrast, the doping concentration in the drift region of the SSJ-UMOS (*N_D_*_,*SSJ*_) is determined by the two-dimensional charge coupling of the SJ structure and MIS structure SIPOS/oxide/Si. To achieve an effective charge-coupling effect, the highly doped N-pillar region with the total charge *Q_N_*_,*SSJ*,*total*_ must be completely depleted by the P-pillar of SJ structure with the charge *Q_SJ_*_,*P*_ and the MIS structure of SIPOS/oxide/Si with the charge *Q_SIPOS_*_,*C*_, as the drain bias approaches the BV, given by

(2) $$Q_{N,SSJ,total}=Q_{P,SJ}+Q_{C,SIPOS}$$

When both the N-pillar and P-pillar are simultaneously depleted, and the E-field at junction *J_SJ_* reaches *E_CU_*, indicating breakdown in the case of a uniform E-field distribution as

(3) $$Q_{\;P,SJ}=q{N\prime }_{D,SJ}W_{N}=qN_{A,SJ}W_{P}=2\varepsilon_{Si}E_{CU}$$

where *N*′_*D*,*SJ*_ represents the equivalent doping concentration for depleting the P-pillar within the N-pillar. *Q_SIPOS_*_,*C*_ denotes the charge of the equivalent plate capacitor for the Si/oxide/SIPOS structure, equating to the partial charge with the equivalent doping concentration of *N*″_*D*,*SJ*_ in the N-pillar as

(4) $$Q_{C,SIPOS}=\Delta V\varepsilon_{Si}L_{D}/t_{OX}=q{N\prime \prime }_{D,SJ}W_{N}L_{D}$$

where Δ*V* represents the potential difference across the thin oxide layers between the SIPOS pillar and the N-pillar. *ε_OX_* and *ε_Si_* denote the permittivity of the oxide and Silicon. *t_OX_* is the oxide thickness. The total doping concentration of the N-pillar due to SIPOS modulation of SSJ-UMOS structure can be obtained as

(5) $$N_{D,SSJ}={N\prime }_{D,SJ}+{N\prime \prime }_{D,SJ}=\varepsilon_{Si}(2t_{OX}E_{CU}+\Delta V)/qW_{N}t_{OX}$$

### 2.2. Electric Field of SIPOS Modulated Drift Region

Assuming a reverse bias *V_R_* is applied, and the drift region is fully depleted, the electrostatic potential *ϕ* must satisfy the following Poisson equation with appropriate boundary conditions [20].

(6) $$\nabla^{2}\phi =\frac{\partial^{2}\phi (x,y)}{\partial x^{2}}+\frac{\partial^{2}\phi (x,y)}{\partial y^{2}}=-\frac{\chi }{\varepsilon_{S}},\;0\leq y\leq L_{D}$$

(7) $$\left\{\begin{array}{l} \chi =qN_{D,total},\;\varepsilon =\varepsilon_{Si}\;\mathrm{when}\;-W_{N}/2\leq x\leq W_{N}/2\\ \chi =0,\;\;\varepsilon =\varepsilon_{ox}\;\mathrm{when}\;W_{N}/2\;<x\leq t_{OX}+W_{N}/2 \end{array}\right.$$

**Figure 2:**
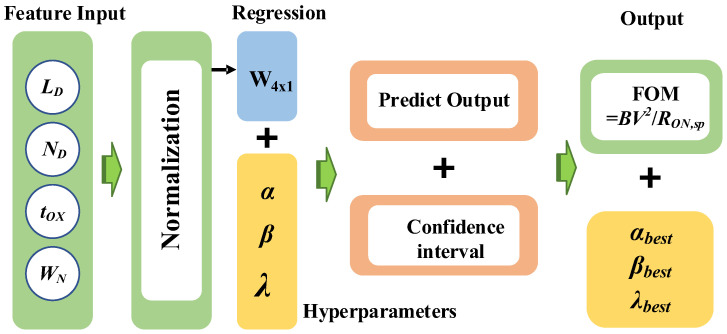
Schematic representation of the Gaussian process regression model.

Considering strong coupling and electric displacement continuity at the semiconductor–dielectric interface, appropriate boundary conditions in the *y*-direction can be established as

(8) $$\left\{\begin{array}{l} {\left.\frac{\partial \phi (x,y)}{\partial x}\right|}_{x=0}=\frac{\partial \phi (0,y)}{\partial x}=E(0,y),\;\vec{E(0,y)}=\vec{E_{L}}\\ {\left.\frac{\partial \phi (x,y)}{\partial x}\right|}_{x=\frac{W}{2}}=E(\frac{W_{N}}{2},y),\;\vec{E(\frac{W_{N}}{2},y)}=\vec{E_{\mathrm{Si-OX},SSJ}}+\vec{E_{L,SSJ}} \end{array}\right.$$

where *E* (0, *y*) represents the vertical E-field along the dotted line A-A′ (*x* = 0, Figure 2), where the lateral E-field component is zero. $\vec{E_{L}}$ represents the vertical potential E-field component generated under the drain bias of *V_R_*. At position *x* = *W_N_*_/2_, the E-field comprises the vertical potential E-field component $\vec{E_{L,SSJ}}$ and the lateral plate capacitive potential E-field component $\vec{E_{\mathrm{Si-OX},SSJ}}$, expressed as

(9) $$\left\{\begin{array}{l} \varepsilon_{Si}E_{\mathrm{Si-OX},SSJ}=\varepsilon_{OX}E_{OX}=\varepsilon_{OX}\cdot \Delta V/t_{OX}\\ E_{\mathrm{Si-OX},SSJ}=\alpha E_{CU},\;(0<\alpha <1)\\ E_{L,SSJ}=\beta E_{CU},\;(0<\beta <1)\; \end{array}\right.$$

where Δ*V* is the potential difference across the thin oxide layers, between the voltage on the N-pillar $\phi_{Si}(y)$ and the voltage on the SIPOS pillar $\psi_{\mathrm{SIPOS}}(y)$. *α* and *β* are coefficients of SSJ-UMOS with values between 0 and 1. The potential in the SIPOS layer is assumed to be linearly distributed in the drift region based on the ohmic behavior of the SIPOS layer as

(10) $$\left\{\begin{array}{l} \Delta V=\phi_{Si}(y)-\psi_{SIPOS}(y)\;,\psi_{SIPOS}(y)=V_{D}(y/L_{D})\\ \phi_{Si}(y=0)=0\;,\phi_{Si}(y=L_{D})=V_{D} \end{array}\right.$$

The potential function is approximated by a second-order Taylor expansion formula. By solving the 2-D Poisson’s equations with the boundary conditions (6)–(8), a general differential equation for the potential distribution function in the N-pillar drift region is obtained as

(11) $$\frac{\partial^{2}\phi (0,y)}{dy^{2}}-\frac{\partial \phi (0,y)}{{T_{S}}^{2}}=-\frac{qN_{eff}}{\varepsilon_{Si}},0\leq y\leq L_{D}$$

where *T_s_* is expressed as

(12) $$T_{S}=\sqrt{\frac{W_{N}}{2}(\frac{W_{N}}{4}+\frac{\varepsilon_{Si}}{\varepsilon_{OX}}t_{OX})}$$

*N_eff_* is the effective doping concentration of the N-drift region. Solving (11) with constraints (8)–(10) gives the potential distributions in the N-pillar as

(13) $$\phi (x,y)=[1-\frac{x^{2}}{2T_{S}}][\frac{qN_{eff}T_{S}^{2}}{\varepsilon_{Si}}-\frac{qN_{eff}T_{S}^{2}}{\varepsilon_{Si}}\frac{\sinh(\frac{y}{T_{S}})+\sinh(\frac{L_{D}-y}{T_{S}})}{\sinh(\frac{L_{D}}{T_{S}})}+V_{D}\frac{\sinh(\frac{y}{T_{S}})}{\sinh(\frac{L_{D}}{T_{S}})}]$$

In the scenario where the E-field extends through the entire length of the drift region, the magnitude of the E-field in the y-direction *E*(*y*) along the middle line of the N-drift region is given by

(14) $$E(0,y)=\frac{V_{D}}{T_{S}}\frac{\cosh(\frac{y}{T_{S}})}{\sinh(\frac{L_{D}}{T_{S}})}+\frac{qN_{eff}T_{S}}{\varepsilon_{Si}}\left[\frac{\cosh(\frac{L_{D}-y}{T_{S}})}{\sinh(\frac{L_{D}}{T_{S}})}-\frac{\cosh(\frac{y}{T_{S}})}{\sinh(\frac{L_{D}}{T_{S}})}\right]$$

For the SSJ-UMOS structure with *N_eff_* = *N*_*D*,*SSJ*_ (5), *E_SSJ_* (*y*) is expressed as

(15) $$E_{SSJ}(y)=\frac{\Delta V+2E_{CU}t_{OX}}{W_{N}t_{OX}}T_{S}e^{-y/T_{S,SJ}}+(\frac{V_{D}}{T_{S}}-\frac{\Delta V+2E_{CU}t_{OX}}{W_{N}t_{OX}}T_{S})e^{(y-L_{D})/T_{S}}$$

Combining (9) an optimum expression for *T_S_* can be derived under the criterion that the E-field at the junction *J_SJ_* and at the bottom of the trench are equal at the breakdown, for the condition as

(16) $$\left\{\begin{array}{l} E(y=0)=E(y=L_{D})=E_{CU}\\ \sqrt{E_{L,SSJ}^{2}+E_{\mathrm{Si-OX},SSJ}^{2}}=E_{CU} \end{array}\right.$$

The *BV* of SSJ-UMOS is expressed as

(17) $$BV=\lambda E_{CU}L_{D},\;0<\lambda <1$$

where *λ* is a coefficient with values between 0 and 1. Combined with the solution of (11), (14), and (16), the optimum *T_S_*_,*OP*_ is given by

(18) $$T_{S,OP}=\sqrt{\frac{\varepsilon_{Si}BV}{2qN_{eff}}}$$

Utilizing Equations (5), (12), and (18), we determine the optimal oxide thickness *t*_*OX*,*OP*_ for SIPOS SJ-UMOS as

(19) $$t_{OX,\;OP}=\frac{\varepsilon_{OX}}{\varepsilon_{Si}}[\frac{BV}{(2+\alpha \varepsilon_{Si}/\varepsilon_{OX})E_{CU}}-\frac{W_{N}}{4}]$$

### 2.3. Figure of Merit BV-R_ON,sp_ Model for SSJ-UMOS

Combining the SJ and MIS structures enables the SSJ-UMOS to achieve ultra-low *R*_*ON*,*sp*_. The total drift region resistance is analyzed in two components: one from the highly doped N-pillar drift region and the other from the carrier accumulation layer due to positive gate bias on the MIS structure SIPOS/oxide/Si. The *R_SJ_*_,*sp*_ contributed by the N-pillar drift region is expressed as

(20) $$R_{SJ,sp}=\rho L_{D}\frac{W_{Cell}}{W_{N}}=\frac{1}{q\mu_{N}N_{eff}}L_{D}\frac{W_{Cell}}{W_{N}}$$

where *W_Cell_* is half the width of the cell (*W_N_* + *W_P_* + *W_I_*). *ρ* is the resistivity of the N-pillar drift region. *μ_N_* is the electron mobility. When (5) and (20) are combined, the *R_SJ_*_,*sp*_ contributed by the N-pillar in SSJ-UMOS is expressed as

(21) $$R_{SJ,sp}=\frac{W_{Cell}L_{D}}{\mu_{N}\varepsilon_{Si}(2t_{OX}E_{CU}+\Delta V/t_{OX})}$$

The schematic cross-section illustrates the SIPOS pillar modulated SJ drift region and the carrier accumulation layer along the trench surface in the N-pillar drift region. Due to the uniform resistivity of the SIPOS layer, the voltage across the SIPOS at position y is denoted as *V*(*y*)

(22) $$V(y)=[(V_{D}-V_{G})/L_{D}]y+V_{G}$$

The specific resistance *R_A_*_,*sp*_ of the accumulation layer is obtained by integrating the *dR*_*A*,*sp*_, is expressed as

(23) $$R_{A,SP}=\int_{0}^{L_{D}}\frac{W_{Cell}}{\mu_{N}C_{OX}(V(y)-V_{th})}\;dy$$

In the ON state, the threshold voltage (*V_th_*) signifies the initiation of the accumulation layer formation. Substituting (22) into (23), we obtain the integrated result for *R_A_*_,*sp*_ as

(24) $$R_{A,SP}=\frac{\ln(V_{D}-V_{th})-\ln(V_{G}-V_{th})}{V_{D}-V_{G}}\frac{W_{Cell,SJ}}{\mu_{N}C_{OX}}L_{D}\;$$

As the total *R_ON_*_,*sp*_ contributed by the drift region and the accumulation layer is in parallel, the overall *R_ON_*_,*sp*,*SSJ*_ for the SSJ-UMOS comprises two components, *R_SJ_*_,*sp*_ and *R_A_*_,*sp*_ as

(25) $$R_{ON,sp,SSJ}=R_{SJ,sp}||R_{A,sp}$$

Combining (21), (24) and (25), the *R_ON_*_,*sp*,*SSJ*_ is obtained as

(26) $$\left\{\begin{array}{l} R_{ON,sp,SSJ}=\frac{W_{Cell}ML_{D}}{\mu_{N}\left[C_{OX}+\varepsilon_{Si}M(2t_{OX}E_{CU}+\Delta V/t_{OX})\right]}\\ M=\frac{\ln(V_{D}-V_{th})-\ln(V_{G}-V_{th})}{V_{D}-V_{G}} \end{array}\right.$$

When applying Baliga’s formula for the impact ionization coefficient, αSi, to a two-dimensional charge-coupling silicon device, as referenced in [19], we derive an expression for the critical electric field in scenarios characterized by a uniform electric field as

(27) $$\left\{\begin{array}{l} \int_{0}^{L_{D}}\alpha_{\mathrm{Si}}\;dx=1,\;\alpha_{\mathrm{Si}}=3.51\times 10^{-35}{E_{CU}}^{7}\\ E_{CU}=8.36\times 10^{4}{L_{D}}^{-1/7} \end{array}\right.$$

When (9), (17), (26), and (27) are combined, the *R_ON_*_,*sp*,*SSJ*_ is given by

(28) $$R_{ON,sp,SSJ}=\frac{W_{Cell}M}{\mu_{N}\left[C_{OX}+\varepsilon_{Si}ME_{CU}(2t_{OX}+\alpha \varepsilon_{Si}/\varepsilon_{OX})\right]}\frac{BV^{7/6}}{5.53\times 10^{5}\lambda^{7/6}}$$

The mobility *μ_N_* is influenced by the silicon-oxide interface property. In practical processes, the SSJ-UMOS resistance is increased due to side-wall mobility degradation. The *R_ON_*_,*sp*,*SSJ*_ surpasses the superjunction UMOS Silicon limit mentioned in Ref. [19], which is given by

(29) $$R_{ON,sp}(\mathrm{Ideal}\;\mathrm{SJ})=\frac{3.27\times 10^{-12}BV^{4/3}W_{N}}{\varepsilon_{S}\mu_{N}}$$

### 2.4. Hyperparameters Optimization Based on Gaussian Process Regression Model

Figure 1b illustrates a phased approach for optimizing hyperparameters (*α*, *β*, *λ*) using Gaussian processes. Figure 2 shows the schematic representation of the GPR. Following device model establishment, we analyze the electrical mechanism and conducted Sentaurus TCAD simulations to generate a dataset containing 1000 samples. Subsequently, GPR is applied to construct a *FOM* = *BV*^2^/*R_ON_*_,*sp*_ prediction model and identify optimal hyperparameters. Structural parameters such as *L_D_*, *N_D_*, *t_OX_*, *W_N_*, closely linked to *FOM*, are considered during hyperparameter optimization. GPR, a non-parametric Bayesian regression method, assumes the target variable *FOM* follows a multivariate Gaussian distribution, avoiding specific assumptions about the fitting function F and treating *FOM* at any data point x as a random variable. Combining (16), (27) and (28), the *FOM* calculation formula is the target formula to be optimized for the GPR model, expressed as

(30) $$\begin{array}{l} FOM=\frac{BV^{2}}{R_{ON,sp,SSJ}}=\frac{6.99\times 10^{9}\mu_{N}\lambda^{2}{L_{D}}^{5/7}\left[C_{OX}+Mt_{OX}[qW_{N}N_{D,SSJ}+\alpha E_{CU}\frac{\varepsilon_{Si}^{2}}{\varepsilon_{OX}}(\frac{1}{t_{OX}}-1)]\right]}{W_{Cell}M},\sqrt{\alpha^{2}+\beta^{2}}\;\leq 1 \end{array}$$

After establishing the device model, TCAD simulations are employed to generate device data for different combinations of *L_D_*, *N_D_*_,*SSJ*_, *t_OX_*, *W_N_*. Subsequent data processing leads to the dataset as

(31) $$D={\left\{(L_{D_{i}},N_{D,SSJ_{i}},t_{ox_{i}},W_{N_{i}},FOM_{i})\right\}}_{i=1}^{N}$$

where *L_Di_*, *N_Di_*, *t_OXi_*, *W_Ni_* denote the features of the *i*-th data point, corresponding to the target value *FOM_i_*, representing the FOM of the *i*-th device.

The mean function *m*(*x*) represents the average behavior of the target value FOM given the features *L_D_*, *N_D_*_,*SSJ*_, *t_OX_*, *W_N_*. The covariance kernel function *k* (*x*, *x*′) represents the correlation between different data points *x* and *x*′ in the feature space as

(32) $$FOM\sim N(m(x),k(x,x^{\prime }))$$

We then define the likelihood function based on the derived (32) to express the probability of observing the data given the parameters *α*, *β*, *λ*. In GPR, the likelihood function is represented using a Gaussian distribution and expressed as

(33) $$L(\alpha ,\beta ,\gamma )=P(\alpha ,\beta ,\gamma )=\prod_{i=1}^{N}P(FOM_{i}|L_{D_{i}},N_{D_{i}},t_{OX_{i}},W_{N_{i}},\alpha ,\beta ,\lambda )$$

For each data point, we employ a multivariate Gaussian distribution as the probability distribution, calculating the mean and variance from the dataset. The likelihood function is obtained through maximum likelihood estimation, and a gradient descent optimization algorithm is applied to optimize the three hyperparameters *α*, *β*, and *λ* resulting in the final values *α*_best_, *β*_best_, and *λ*_best_.

## 3. Results and Discussion

### 3.1. Off-State Characteristics

**Figure 3:**
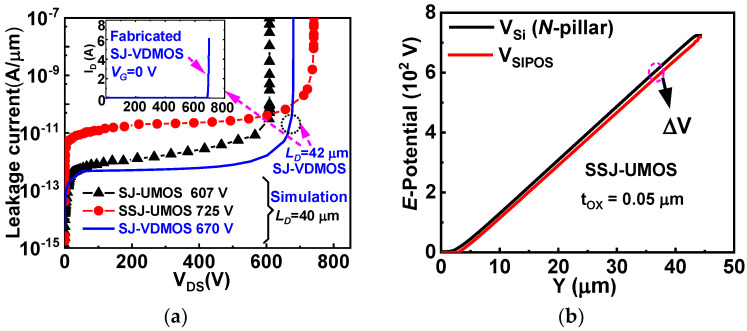
(**a**) Reverse leakage current versus V_DS_ of SSJ-UMOS, SJ-UMOS, and simulation results calibrated to breakdown characteristics (I_DS_-V_DS_) data from the fabricated SJ-VDMOS [21]. (**b**) Electric potential difference (Δ*V*) between the drift region and the SIPOS pillar of SSJ-UMOS.

Numerical results obtained through TCAD simulations and analytical results from the model are compared. To validate the model, simulation results are calibrated to breakdown characteristic (I_D_-V_D_) data extracted from fabricated SJ-VDMOS [21], as depicted in Figure 3a. The TCAD simulation results, with a single set of self-consistent parameters, align well with experimental data. Additionally, the OFF state characteristics of SJ-UMOS and SSJ-UMOS are illustrated in Figure 3a. As the resistivity of the SIPOS layer equals 1.0 × 10^10^ Ω·cm, the leakage current of SSJ-UMOS increases from 10^−12^ to 10^−10^ A due to the SIPOS field plate acting as a high-resistor parallel to the drift region. In the OFF state, there is a uniform potential difference (Δ*V*) between the SIPOS layer and the vertical surface of the N-drift region for and SSJ-UMOS, as shown in Figure 3b.

**Figure 4:**
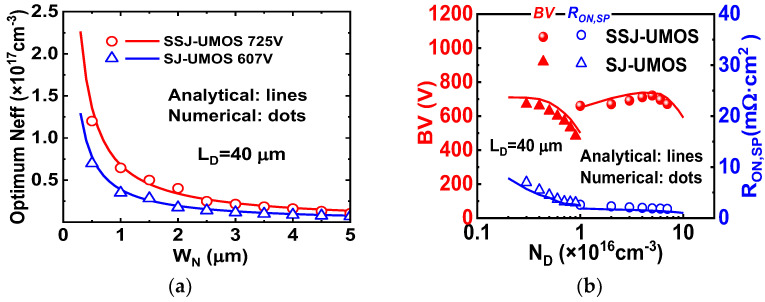
(**a**) Optimum doping concentration for two-dimensional charge-coupling, and (**b**) dependence of BV and *R_ON_*_,*sp*_ on *N_D_* for SSJ-UMOS and SJ-UMOS.

Figure 4a shows the optimum effective doping concentration (*N_eff_*) predicted by expressions (5), (18), and (19) as a function of the *W_N_* with the BV as a parameter. Notably, the optimum dose decreases with increasing *W_N_*. SSJ-UMOS exhibits a higher optimum *N_eff_* than SJ-UMOS, attributed to the enhanced charge coupling effect of SIPOS pillars. In Figure 4b, the dependence of BV and *R_ON_*_,*sp*_ on *N_D_* for SSJ-UMOS and SJ-UMOS is illustrated. In SSJ-UMOS, SIPOS-assisted depletion of N-pillars reduces *R_ON_*_,*sp*_ and increases BV. Compared to SJ-UMOS, the BV of SSJ-UMOS decreases gradually when doping concentration is imbalanced, owing to the E-field modulation of SIPOS pillars.

### 3.2. Gaussian Process Regression

The Gaussian process regression model exhibits exceptional performance in this study. Key evaluation metrics, as shown in Table 1, include a mean squared error (MSE) of 953.56, a root mean squared error (RMSE) of 30.88, and a mean absolute percentage error (MAPE) of only 4.5%. These metrics unequivocally attest to the model’s exceptional predictive accuracy.

**Figure 5:**
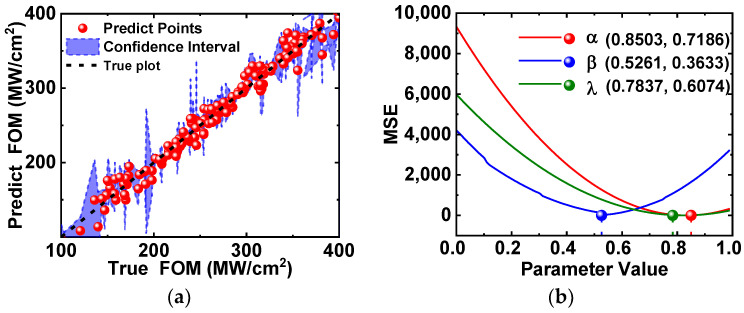
(**a**) Fitting performance of Gaussian process regression model on FOM. (**b**) Hyperparameter sensitivity analysis of α, β and λ.

These results highlight the Gaussian process regression model’s reliability in fitting and prediction, underscoring the crucial role of parameter optimization in enhancing model performance. We utilized visual representations to showcase the model’s performance. In Figure 5a, a confidence interval plot illustrates the model’s precision in predicting the target variable and the associated uncertainty. The model demonstrates low uncertainty, indicating high reliability in predictions, especially near the forecasted values. Figure 5b presents the results of parameter sensitivity analysis, revealing optimal hyperparameters: α = 0.8503, β = 0.5261, and λ = 0.7837. Notably, α significantly influences fitting results, highlighting its sensitivity. This insight provides valuable guidance for further parameter optimization, with the potential to improve both fitting quality and predictive accuracy.

**Figure 6:**
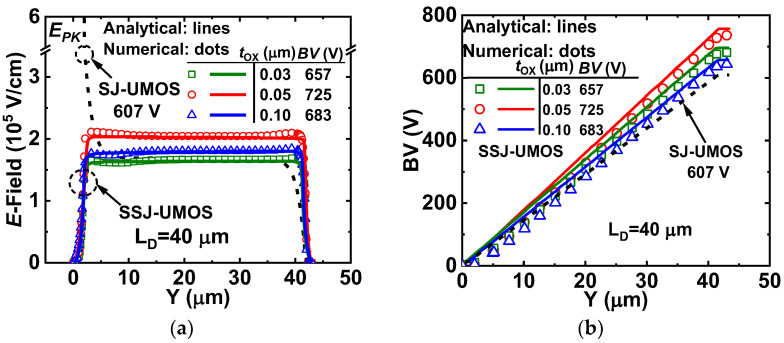
Simulated and analytical (**a**) E-field, and (**b**) potential distributions of SIPOS SJ UMOS and SIPOS UMOS (along the line A-A’).

Analytical expression (15) is applicable in SSJ-UMOS with modifications to *N_eff_* based on Equations (5) and (7). In Figure 6a,b, numerical and analytical profiles of vertical E-field and potential for SSJ-UMOS and SJ-UMOS in the middle of the N-pillar along the y-direction (A-A’, Figure 1a) are presented. In comparison to SJ-UMOS without the SIPOS layer, the high E-field peak (E_PK_) at the gate trench bottom is reduced and BV is improved from 607 V to 725 V. Analytical results for SSJ-UMOS align with numerical results for various *T_OX_* values. Optimizing the oxide layer thickness (*T_OX_* = 0.05 μm) in SSJ UMOS effectively enhances device performance.

**Figure 7:**
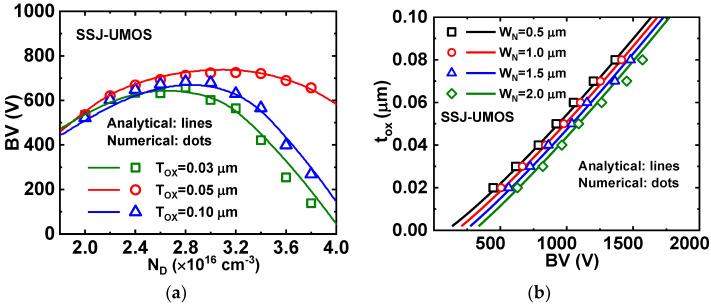
Optimum (**a**) oxide thickness t_OX_ and (**b**) N-pillar width *W_N_* for SSJ UMOS.

Figure 7a,b present the optimum oxide thicknesses and N-pillar width for SSJ-UMOS with various breakdown voltages, as predicted by the analytical model (18) and (19). The trench oxide thickness increases for devices with larger blocking voltages, staying within practical limits for device processing and fabrication. For a breakdown voltage of 1000 V, the optimal trench oxide thickness is 0.05 μm with a mesa width of 1.0 μm for SSJ-UMOS, aligning with the obtained numerical results.

### 3.3. ON State and Dynamic CHARACTERISTIC

**Figure 8:**
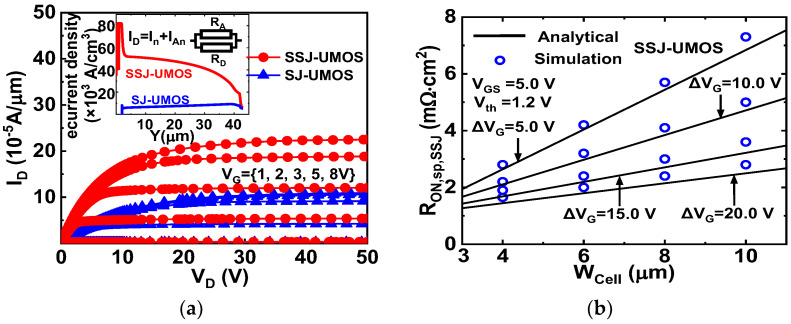
(**a**) Electron current density distribution and output characteristics for SSJ-UMOS and SJ-UMOS. (**b**) Simulated and analytical *R_ON_*_,*sp*,*SSJ*_ at the different Δ*V_G_* and *W_Cell_* for the SSJ-UMOS.

Figure 8a displays electron current density distributions in the drift region and output characteristics of SSJ-UMOS and SJ-UMOS. The threshold voltage *V_th_* of the two devices are about 1.2 V. In SSJ-UMOS, drift region resistance (*R_D_*) is the parallel connection of the accumulation layer resistance (*R_A_*). The maximum electron current density of SSJ UMOS reaches 8.23 × 10^4^ A/cm^2^, significantly higher than that of SJ UMOS. At a high drain voltage, the second term M in (26) becomes dominant, leading to a strong dependence on Δ*V_G_* = *V_D_* − *V_G_* as shown in Figure 8b. Additionally, reducing the pitch *W_cell_* can decrease *R_ON_*_,*sp*,*SSJ*_.

**Figure 9:**
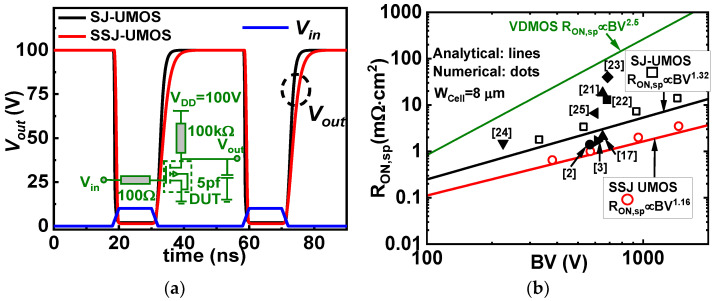
(**a**) Switching waves of SIPOS SJ-UMOS and the conventional SJ-UMOS at the same V_DD_ = 100 V. (**b**) Comparison of theoretical predictions of *R_ON_*_,*sp*_ versus BV relationship of SSJ-UMOS, SJ-UMOS and other published devices with the ideal silicon limit and the SJ silicon limit line in the BV range of 10–1000 V.

Figure 9a presents a dynamic performance comparison between SIPOS SJ-UMOS and the conventional SJ-UMOS. The SIPOS pillars increase gate capacitance, generating a surface deep-depletion layer in the drift region in the OFF state, leading to switching delays in SIPOS SJ-UMOS. The turn-on speed is comparable between the two devices, while the turn-off speed of SIPOS SJ-UMOS is slower than that of the conventional model. Nonetheless, MOSFETs with SIPOS terminations have demonstrated resilience under harsh conditions, such as a gradient of 10 kV/μs. In Figure 9b, a comparison of the *R_ON_*_,*sp*_ and BV relationships is presented for the three structures, including references [2,3,17,21,22,23,24,25]. Optimum *t_OX_*, *W_N_*, *L_D_*, and *N_D_* values for SSJ-UMOS and S-UMOS are chosen for this analysis. For the *R_ON_*_,*sp*_ analysis of SSJ-UMOS, V_DS_ is set to 10 V at V_GS_ of 5 V. The plot in Figure 9b clearly indicates that the SSJ-UMOS structure outperforms other structures, surpassing the SJ silicon limit [19].

## 4. Conclusions

This paper introduces a machine learning-based figure of merit model of SSJ-UMOS featuring a modulated drift region utilizing SIPOS pillars. The tradeoff characteristics between BV and *R_ON_*_,*sp*_ have been theoretically derived, breaking the SJ Silicon limit by applying three methods for the additional E-field modulation effect, charge coupling effect and majority carrier accumulation, simultaneously. In the analytical model, the optimal structure parameters of the drift region, oxide thickness, and E-field modulation coefficients are also discussed in the analytical model. GPR is employed for an accurate figure of merit prediction and hyperparameter optimization, which can give guidance for the design of power MOSFETs with SIPOS. The proposed model’s validity is robustly confirmed through comprehensive verification against TCAD simulation results.

## Figures and Tables

**Table 1:** Model Fitting Evaluation Metrics.

Evaluation Metric	Metric Value
MSE	953.56
RMSE	30.88
MAPE	4.5%

## Data Availability

The original contributions presented in the study are included in the article, further inquiries can be directed to the corresponding author.

## References

[1] Udrea F., Deboy G., Fujihira T. (2017). Superjunction power devices, history, development, and future prospects. IEEE Trans. Electron Devices.

[2] Wu L., Chen X., Zeng J. (2022). Novel accumulation mode superjunction device with extended superjunction gate. IEEE Trans. Electron Devices.

[3] Duan B., Wang Y., Sun L. (2020). Accumulation-mode device: New power MOSFET breaking superjunction silicon limit by simulation study. IEEE Trans. Electron Devices.

[4] Saito W. (2018). Breakthrough of drain current capability and on-resistance limits by gate-connected superjunction MOSFET. Proceedings of the 2018 IEEE 30th International Symposium on Power Semiconductor Devices and ICs (ISPSD).

[5] Williams R.K., Darwish M.N., Blanchard R.A. (2017). The trench power MOSFET: Part I—History, technology, and prospects. IEEE Trans. Electron Devices.

[6] Luo X., Jiang Y.H., Zhou K. (2012). Ultralow specific on-resistance superjunction vertical DMOS with high-K dielectric pillar. IEEE Electron Device Lett..

[7] Guo Y., Yao J., Zhang B. (2015). Variation of lateral width technique in SoI high-voltage lateral double-diffused metal–oxide–semiconductor transistors using high-k dielectric. IEEE Electron Device Lett..

[8] Cao Z., Wang Q., Jiao L. (2021). Analytical study on a 700 V triple RESURF LDMOS with a variable high-K dielectric trench. IEEE Trans. Electron Devices.

[9] Cao Z., Duan B., Shi T. (2017). A superjunction U-MOSFET with SIPOS pillar breaking superjunction silicon limit by TCAD simulation study. IEEE Electron Device Lett..

[10] Li X., Wu Z., Rzepa G., Karner M., Xu H., Wu Z., Wang W., Yang G., Luo Q., Wang L. (2024). Overview of Emerging Semiconductor Device Model Methodologies: From Device Physics to Machine Learning Engines. Fundam. Res..

[11] Ghoshhajra R., Biswas K., Sarkar A. A review on machine learning approaches for predicting the effect of device parameters on performance of nanoscale MOSFETs. Proceedings of the 2021 Devices for Integrated Circuit (DevIC).

[12] Klemme F., Prinz J., Van Santen V.M. Modeling emerging technologies using machine learning: Challenges and opportunities. Proceedings of the 39th International Conference on Computer-Aided Design.

[13] Wang J., Kim Y.H., Ryu J. (2021). Artificial neural network-based compact modeling methodology for advanced transistors. IEEE Trans. Electron Devices.

[14] Xu C., Liu Y., Liao X. (2021). Machine Learning Regression-Based Single-Event Transient Modeling Method for Circuit-Level Simulation. IEEE Trans. Electron Devices.

[15] Zhang L., Chan M. (2017). Artificial neural network design for compact modeling of generic transistors. J. Comput. Electron..

[16] Mehta K., Wong H.Y. (2020). Prediction of FinFET current-voltage and capacitance-voltage curves using machine learning with autoencoder. IEEE Electron Device Lett..

[17] Zhang W., Zhang B., Li Z. (2015). Theory of superjunction with NFD and FD modes based on normalized breakdown voltage. IEEE Trans. Electron Devices.

[18] Fujihira T. (1997). Theory of semiconductor superjunction devices. Jpn. J. Appl. Phys..

[19] Baliga B.J. (2010). Advanced Power MOSFET Concepts.

[20] Zhou J., Huang C.F., Cheng C.H. (2016). A comprehensive analytical study of dielectric modulated drift regions—Part I: Static characteristics. IEEE Trans. Electron Devices.

[21] Ye Z.Y., Liu L., Yao Y. (2019). Fabrication of a 650V superjunction MOSFET with built-in MOS-channel diode for fast reverse recovery. IEEE Electron Device Lett..

[22] Saito W., Omura I., Aida S. (2006). A 15.5 m Ω cm^2^-680V Superjunction MOSFET Reduced On-Resistance by Lateral Pitch Narrowing. Proceedings of the 2006 IEEE International Symposium on Power Semiconductor Devices and IC’s.

[23] Kushwaha P.K., Nautiyal P., Gupta A. (2019). An improved SJ UMOS with modified gate electrode to reduce gate charge. Proceedings of the 2019 9th Annual Information Technology, Electromechanical Engineering and Microelectronics Conference (IEMECON).

[24] Shibata T., Noda Y., Yamauchi S. (2007). 200V trench filling type super junction MOSFET with orthogonal gate structure. Proceedings of the 19th International Symposium on Power Semiconductor Devices and IC’s.

[25] Lin Z., Huang H., Chen X. (2014). An improved superjunction structure with variation vertical doping profile. IEEE Trans. Electron Devices.

